# Supplementary material for: Optical patterning of trapped charge in nitrogen-doped diamond
Source: Nat Commun. 2016 Aug 30;7:12660. doi: 10.1038/ncomms12660 (PMC5013603; doi:10.1038/ncomms12660)
Supplement: Supplementary Information — Supplementary Figures 1-7, Supplementary Table 1, Supplementary Notes 1-2 and Supplementary References. [file ncomms12660-s1.pdf]

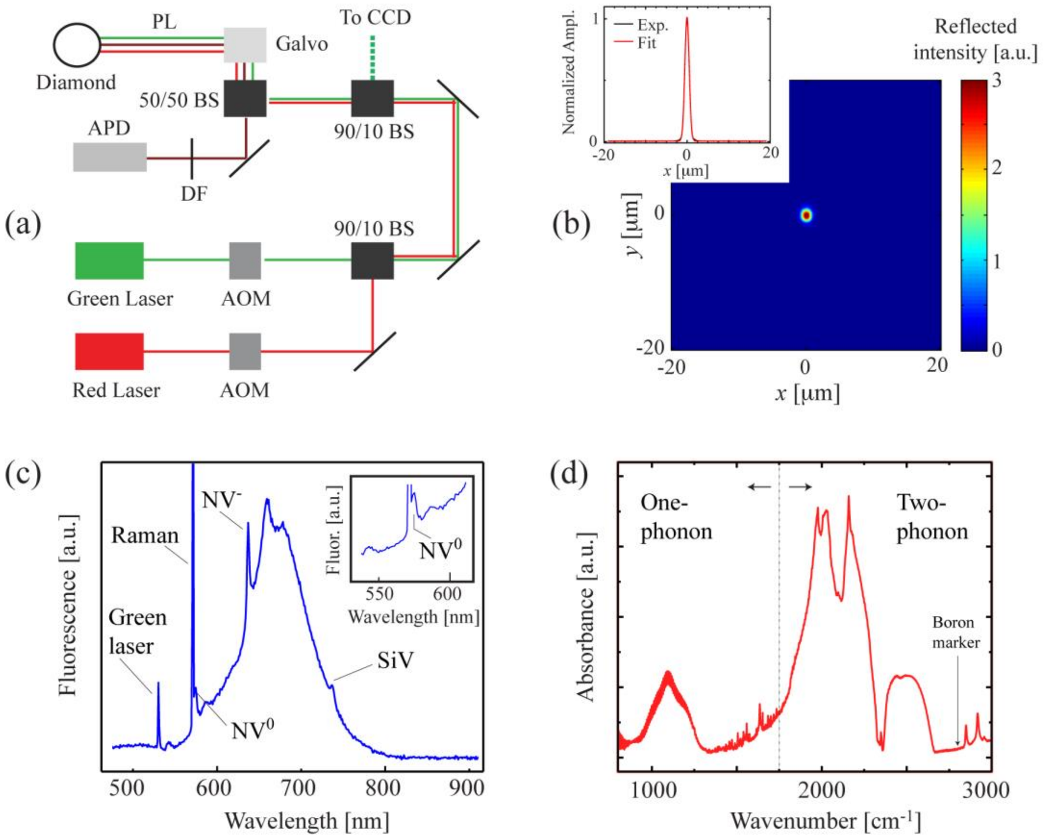

Supplementary Fig. 1 | Experimental setup and additional sample characterization. (a) Schematics of the two-color confocal microscope. To image the beam shape, the green beam is redirected so as to bypass the galvo and focused on a mirror located at the sample position. (b) Scanned image of the fixed green beam upon reflection on the mirror. The shape corresponds to a Gaussian with 1 μm full width at half maximum (FWHM). (c) Typical fluorescence spectrum from the sample diamond crystal. The peak at 737 nm exposes the presence of silicon-vacancy centers at a fractional concentration of 0.6% relative to the NVs. The narrow peak at 532 nm originates from the excitation laser (leaking through the notch filter). (d) Sample infrared spectrum. Using the method of Supplementary Ref. [1], we determine the nitrogen concentration to be 40 ppm. The black arrow highlights the IR amplitude at 2802 cm^{-1}, a marker frequency for boron. Since no peak is observable at this wave number, we conclude no boron is present in our sample. APD: Avalanche photo-detector; AOM: Acousto-optic modulator; CCD: Charge-coupled device; BS: Beam splitter; DF: Dichroic filter.

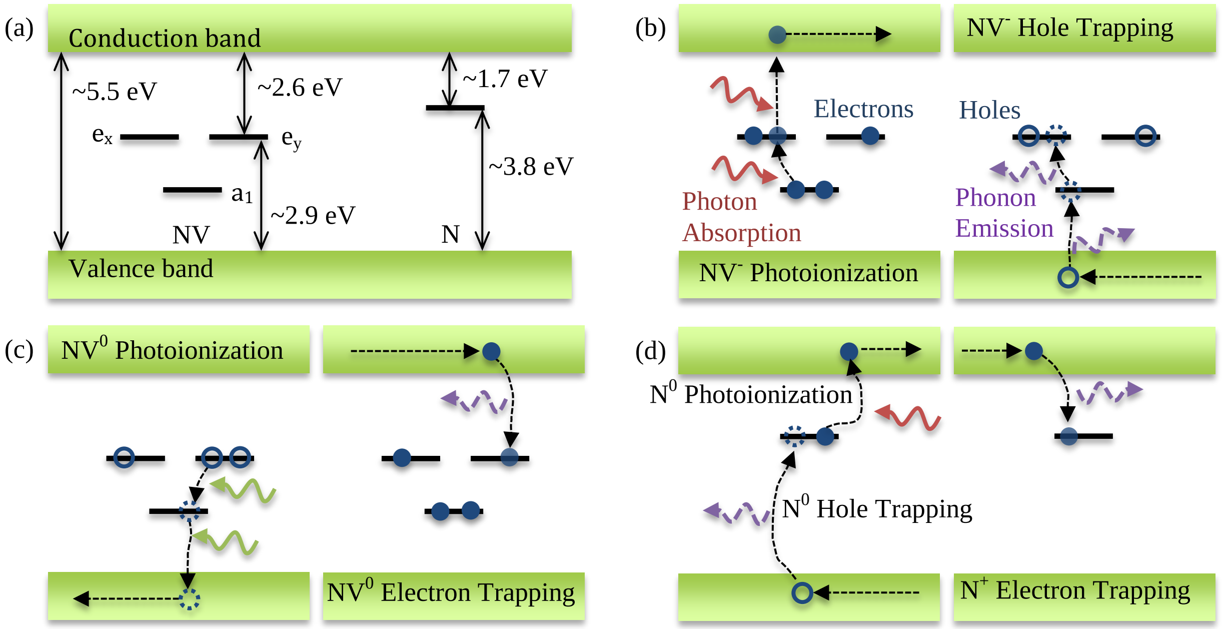

Supplementary Fig. 2 | Dynamics of charge ionization and trapping by NV centers and nitrogen. (a) The defect levels of the NV and N centers within the diamond bandgap. (b)-(d) The NV and N photoionization and carrier trapping mechanisms as described in the text. Symbols are as denoted. Solid (open) circles represent electrons (holes). Red (green) wavy arrows indicate red (green) photons and blue wavy arrows indicate phonons.

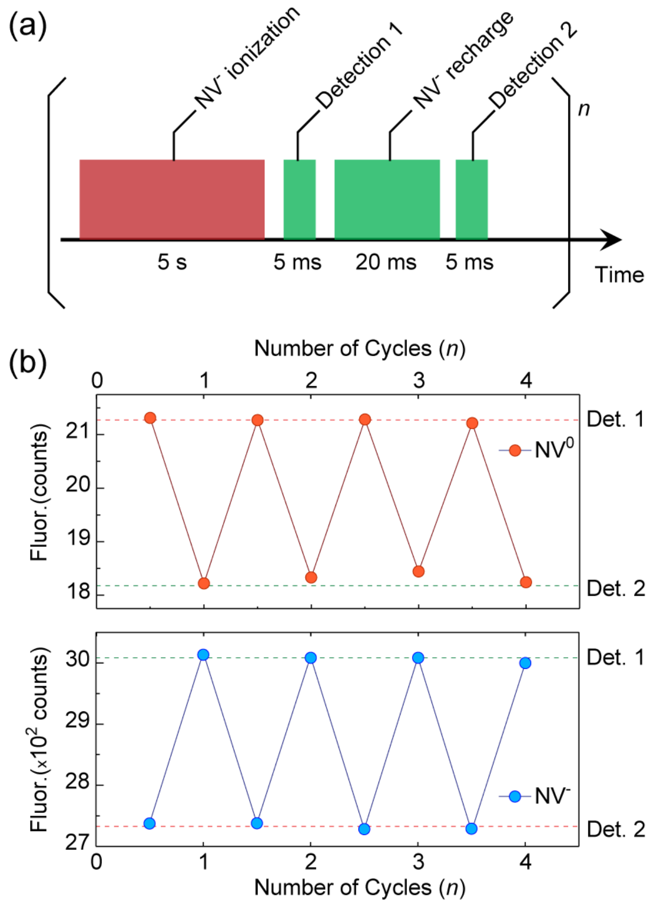

Supplementary Fig. 3 | Evidence for NV charge conversion. (a) Experimental protocol. After a 5 s exposure to red light (1 mW), we detect NV fluorescence during two 5-ms-long pulses of green light (0.5 mW) separated by a 20-ms-long interval of green laser illumination (0.5 mW). The process is repeated *n* times. (b) Integrated NV fluorescence during Detection 1 and Detection 2 (denoted Det. 1 and Det. 2, respectively) as a function of the number of cycles *n*. To isolate contributions from NV^0 (top) or NV^- (bottom), we use a narrow bandpass filter (limited to the range 605-615 nm) or a long pass filter (>637 nm), respectively. The total number of averages per point in the upper (lower) graph is 470 (30). During each cycle the NV^- content grows as the NV^0 concentration shrinks, strongly suggesting green-induced conversion from NV^0 into NV^-.

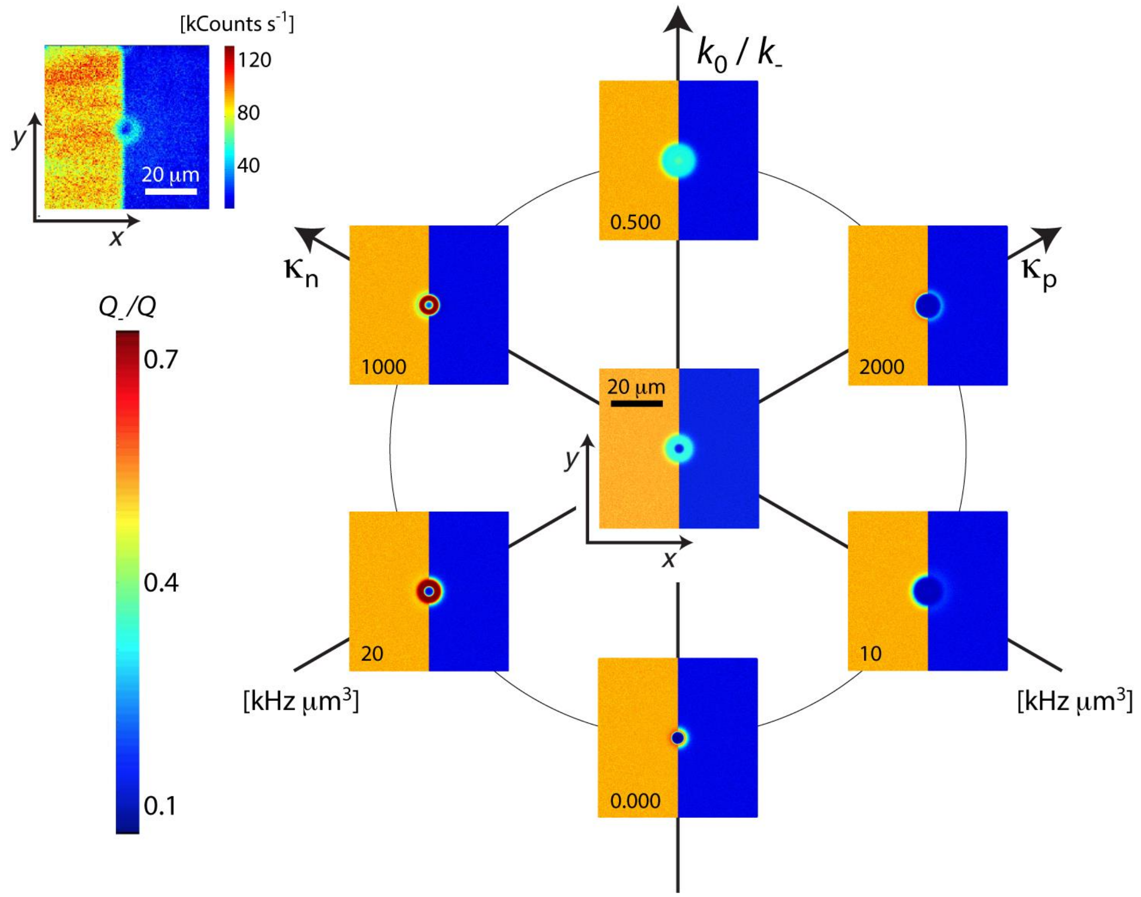

Supplementary Fig. 4 | Parameter estimation under fixed-point red illumination. We compare calculated (center) and experimental (upper left side) NV^- patterns upon 10 s of red light fixed-point excitation (see also Fig. 4 in the main text). For simplicity, we limit the comparison to three independent parameters, namely, the ratio between the $NV^0 \rightarrow NV^-$ and $NV^- \rightarrow NV^0$ conversion rates $(k_0/k_-)|_{632\ \mathrm{nm}}$, and the NV^0 and NV^- electron and hole trapping rates, κ_n and κ_p , respectively. In the central image, all parameters take the values listed in Supplementary Table 1; in the peripheral images the parameter labeling the corresponding axis is altered to take the value listed on the lower left corner of each graph. The scale bar (20 μm) and axes labels are the same for all images.

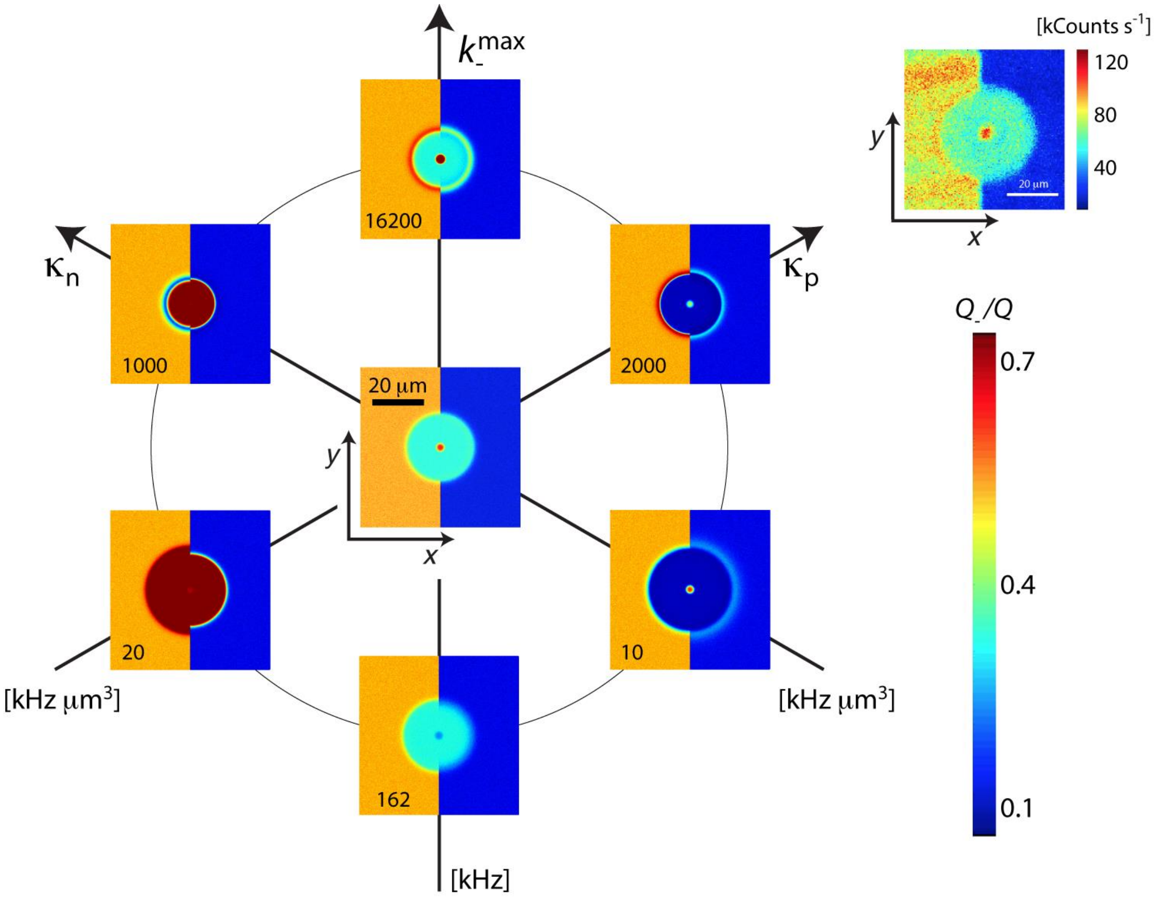

Supplementary Fig. 5 | Parameter estimation under fixed-point green illumination. Similar to Supplementary Fig. 4, we compare calculated and experimental (top central plot) NV^- patterns upon 10 s of green light fixed-point excitation. Once again, we limit our analysis to three independent parameters, including the NV^- photoionization rate k_- at 532 nm, and the NV^0 and NV^- electron and hole trapping rates, κ_n and κ_p, respectively. At the center of the multi-axis figure, all parameters take the values listed in Supplementary Table 1; in the peripheral images the parameter associated to each axis is altered to take the value listed on the lower left corner of each graph while all other parameters remain fixed. The scale bar (20 µm) and axes labels are the same for all images.

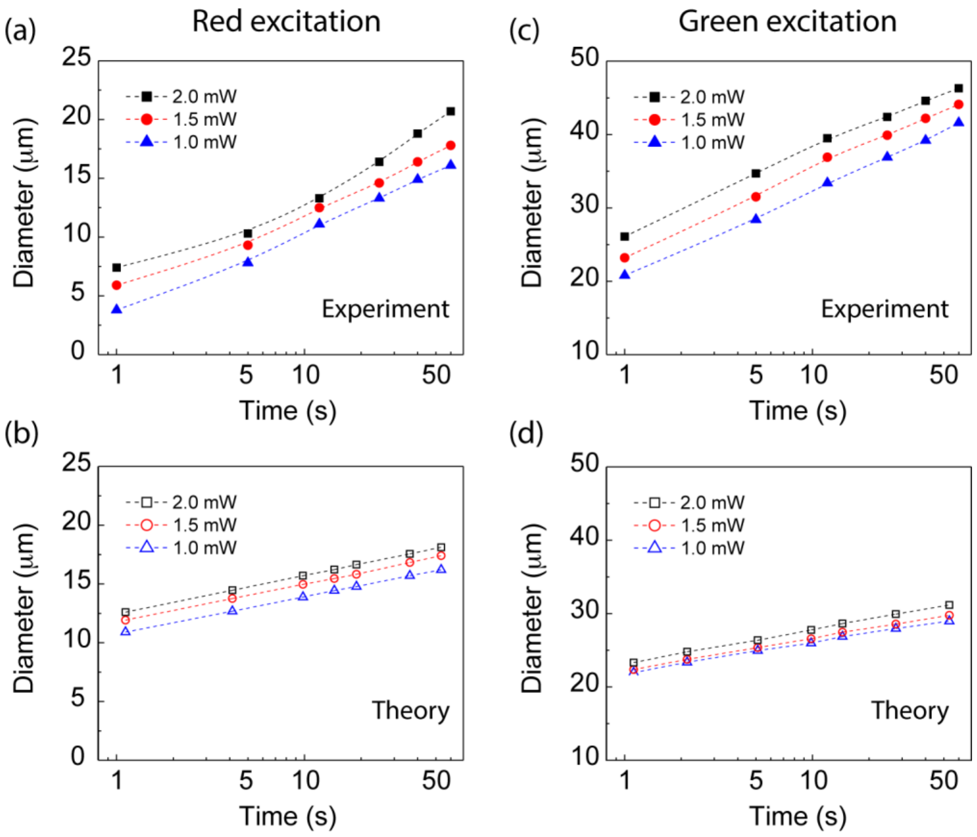

Supplementary Fig. 6 | NV⁻ pattern growth for different laser intensities. (a,b) Experimental (a) and calculated (b) outer radii in the NV⁻ patterns resulting from a red laser park (see also Fig. (2) in the main text). (c,d) Same as in (a,b) but for a green laser park on an unbleached background (see also Fig. 5 in the main text). In all cases, the resulting pattern (not shown for brevity) is imaged via a red laser scan with center at the point of the laser park; the red laser power during the scanning is 2 mW, and the integration time per pixel is 1 ms. Numerical modeling is carried out using the conditions listed in Supplementary Table 1.

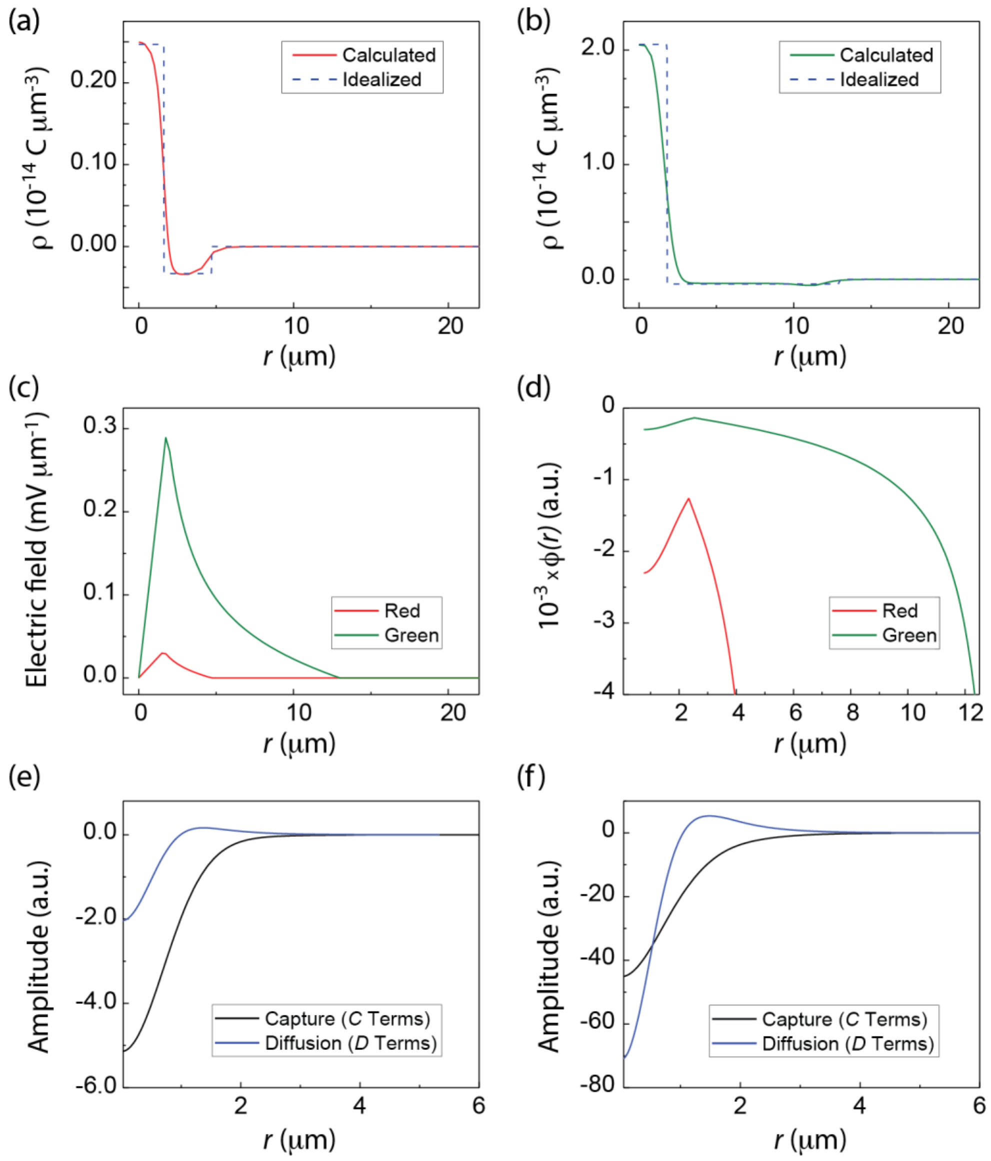

Supplementary Fig. 7 | The influence of electrostatic fields on the transport dynamics of charge carriers. (a,b) Calculated charge density ρ after 10 s of red (a) and green (b) illumination. Dashed lines indicate idealized radial distributions used to simplify the numerical calculation of the electric field map. (c) Calculated electric fields resulting from the simplified charge distributions in (a,b). Given the charge map symmetry, the electric field points in the radial direction. (d) Ratio $\phi(r)$ (see Supplementary Eq. (14)) between diffusion and drift terms in Supplementary Eq. (7) for the case of electrons. Since everywhere in the sample $|\phi(r)| > 1$ (the minimum magnitudes are 1262 and 137 for red and green excitation, respectively), we conclude electrostatic fields do not have a major impact on the carrier dynamics for the present experimental conditions. (e,d) Comparison of the diffusion (D) and capture (C) terms in Supplementary Eq. (10) ($D \equiv D_{\mathrm{n}}\nabla^2 n$ and $C \equiv k_{\mathrm{N}}(P - P_+) - \kappa_{\mathrm{n}} n(Q - Q_-) - \gamma_{\mathrm{n}} n P_+$, respectively) for electrons after 10 s of red (e) or green (f) excitation (see subsection (iv) in Supplementary Note 2).

Parameter	Description	Numerical value	Units	Ref.
$2.36\,\sigma$	Laser beam FWHM<br>@632 nm<br>@532 nm	1.0<br>1.0	µm	[*]
$k_{-}(r)$	Photoionization rate of NV^-<br>@632 nm<br>@532 nm	$(3.6^{+12}_{-3}) \times \left(\frac{I}{I_0}\right)^2 e^{\frac{-r^2}{\sigma^2}}$<br>$(1620^{+8000}_{-1000}) \times \left(\frac{I}{I_0}\right)^2 e^{\frac{-r^2}{\sigma^2}}$	kHz	[†]
$k_0(r)$	Hole production rate from NV^0<br>@632 nm<br>@532 nm	$(0.18^{+0.09}_{-0.09}) \times \left(\frac{I}{I_0}\right)^2 e^{\frac{-r^2}{\sigma^2}}$<br>$(3480^{+15000}_{-2500}) \times \left(\frac{I}{I_0}\right)^2 e^{\frac{-r^2}{\sigma^2}}$	kHz	[†]
$k_{\mathrm{N}}(r)$	Photo-ionization rate of N^0<br>@632 nm<br>@532 nm	$(2.7^{+5}_{-2}) \times 10^{-5} \times \frac{I}{I_0} e^{\frac{-r^2}{2\sigma^2}}$<br>$(27^{+40}_{-10}) \times \frac{I}{I_0} e^{\frac{-r^2}{2\sigma^2}}$	kHz	[†]
κ_{n}	Electron capture rate by NV^0	100^{+50}_{-50}	kHz µm^3	[†]
γ_{n}	Electron capture rate by N^+	300	kHz µm^3	[10,11,14]
κ_{p}	Hole capture rate by NV^-	200^{+100}_{-100}	kHz µm^3	[†]
γ_{p}	Hole capture rate by N^0	0.6	kHz µm^3	[10,11,14]
D_{n}	Electron diffusion constant in diamond	5.5	µm^2 ns^{-1}	[11]
D_{p}	Hole diffusion constant in diamond	4.3	µm^2 ns^{-1}	[11]
Q	NV density	0.4	ppm	[*], [13]
P	nitrogen density	40	ppm	[*]
**Initial conditions**				
Fraction of NV^- centers after red-induced bleaching		0.07		
Fraction of NV^- centers after a green-laser scan		0.5		

Supplementary Table 1 | Summary of simulation parameters. The notation follows that introduced in Eqs. (1) through (4) in the main text; I_0 indicates a reference intensity (2 mW and 1.8 mW for red and green illumination, respectively) used to scale photo-ionization rates at arbitrary illumination intensities I, and r is the distance to the beam center. To reduce the number of free parameters, we use literature values whenever possible. In the last column the number in brackets indicates the corresponding Supplementary Reference, [†] denotes free parameters in our model whose final value is derived from a comparison with the experiment, and [*] indicates a value derived from our sample or setup characterization measurements. All numerical calculations reported herein use the main numerical values listed on the table. See Subsections (ii) and (iii) in Supplementary Note 2 for a description on how to interpret the error margins of free parameters.

Supplementary Note 1 | Model of photoionization and charge diffusion in diamond

In this section, we provide an in-depth description of the theoretical model used to interpret and simulate our defect photoionization and charge diffusion observations. We begin by presenting the relevant defects in type-1b diamond and deriving the equations of their charge state dynamics. We next introduce carrier diffusion in diamond and combine the carrier diffusion equations with the equations of defect dynamics to form the complete model. We finally define the necessary initial and boundary conditions to solve the equations of the model.

i-Defects in type-1b diamond

The three most prevalent defects in type-1b diamond are the nitrogen-vacancy (NV), substitutional nitrogen (N) and isolated vacancy (V) centers.

The NV center is a point defect comprised of a substitutional nitrogen-carbon vacancy pair orientated along the ⟨111⟩ crystallographic direction[2]. It can be stable in both neutral (NV^0) and negative (NV^-) charge states. The stable charge state in thermal equilibrium is determined by the local density of electron donors and acceptors (usually the N center).[2] The NV charge states are characterized by their optical zero-phonon lines (ZPLs) at 2.156 eV (NV^0) and 1.946 eV (NV^-) and their observable electronic structures are defined by the occupation of the center's three deep-level defect orbitals (a_1, e_x, e_y) by three electrons / three holes (NV^0) and four electrons / two holes (NV^-), respectively (Supplementary Fig. 2).

Under green (532 nm) or red (632 nm) illumination, NV^- can be photoionised into NV^0 by ejecting an electron into the diamond conduction band via the successive absorption of two photons[3,4]. In this mechanism, the first photon excites NV^- to its optical excited state and the second photon ejects the electron into the conduction band within the lifetime of the optically excited state. Under green illumination, NV^0 can be photoionized back to NV^- by a similar mechanism involving the ejection of a hole into the diamond valence band and the successive absorption of two photons[3,4] (see Supplementary Fig. 2). The photoionization cross-sections of neither NV charge state are known precisely. Indicative photoionization rates for particular optical powers and focusing arrangements can be drawn from single center studies, which have measured the NV^- photoionization rate to be ~5 Hz under ~1 μW (confocal) of red excitation and the NV^- and NV^0 photoionization rates to be ~25 Hz and ~60 Hz, respectively, under the same power of green excitation[4].

The NV center may also trap charge carriers: NV^- is an attractively charged trap for holes and NV^0 is a neutral trap for electrons. Since both charge states are deep traps, their trapping mechanisms are likely to involve multi-phonon emission[5] (see Supplementary Fig. 2). However, the trapping cross-sections of the NV center have not yet been investigated and their values are unknown.

The N center is a deep donor in diamond and is known to exist in neutral (N^0) and positive (N^+) charge states. There is disagreement about the energy of the N donor level below the conduction band, with various photoabsorption and photoconductivity measurements reporting the energy in the broad range 1.7-2.2 eV.[6-9] We therefore expect that N^0 will photoionize under either green or red excitation. However, we do not expect N^+ will photoionize back to N^0 by the ejection of a hole into the valence band under green or red excitation because this requires much higher energy photons (with energy greater than ~3.8 eV, see Supplementary Fig. 2). Similar to the NV center, the photoionization cross-sections of N^0 are not precisely known. The photoconductivity measurements simply indicate that it is larger under green excitation than under red.

N^0 and N^+ are known to act as deep neutral and attractively charged traps of holes and electrons, respectively. The electron trapping cross section of N^+ has been measured to be in the range ~3-7x10^{-6} μm^2 and the hole trapping cross-section of N^0 has been measured to be ~1x10^{-8} μm^2.[10,11]

The V center is a deep defect in diamond that can exist in neutral V^0 and negative V^- charge states. V^0 and V^- are characterized by the GR1 (with ZPL at 1.673 eV) and ND1 (with ZPL at 3.149 eV) photoabsorption bands, respectively[12]. The GR2-8 bands, which are in the energy range 2.881-3.007 eV, are also attributed to the V^0 center[12]. Photoconductivity studies have revealed that V^0 is photoionized into V^- by ejecting a hole into the valence band if one of the GR2-8 bands are excited, and that V^- is photoionized into V^0 by ejecting an electron into the conduction band if the ND1 band is excited[12]. Consequently, neither V charge state is photo-ionized under red or green excitation. Similar to the NV center, V^0 and V^- are deep neutral electron and attractively charged hole traps, respectively. To our knowledge, the trapping cross-sections of the V center have not yet been determined. Our photoluminescence measurements suggest that there is negligible concentration of V^0 centers in our sample (no GR1 peak is observable at 741 nm in the fluorescence spectrum of Supplementary Fig. 1c) and so the V

center will not be included in our model.

To confirm the transformation of NV^- into NV^0 upon red illumination we alter our confocal microscope to separately collect the fluorescence from two complementary spectral windows. The first one, ranging from 605 nm to 615 nm is exclusively associated with NV^0 emission whereas the second one, from 637 nm to 850 nm, is mostly sensitive to NV^- fluorescence. The experiment of Supplementary Fig. 3 basically reproduces the protocol of Fig. 2 in the main text except that a weak green laser — exciting both NV^- and NV^0 — is used to probe the resulting NV composition. The green laser intensity and exposure time is brought to a minimum so as to limit $NV^-\leftrightarrow NV^0$ inter-conversion. As expected, photon collection from either spectral band leads to complementary NV signatures, with maximum NV^0 fluorescence when NV^- emission is minimum.

ii-Defect dynamics

The coupled dynamics of the charge state densities of NV and N centers as functions of position $\boldsymbol{r}$ and time t are described by the four master equations

$$\frac{\partial Q_-}{\partial t} = k_0 Q_0 - k_- Q_- + \kappa_{\mathrm{n}} n Q_0 - \kappa_{\mathrm{p}} p Q_- + \Omega(Q_0 P_0 - Q_- P_+) = -\frac{\partial Q_0}{\partial t}, \quad (1)$$

$$\frac{\partial P_+}{\partial t} = k_{\mathrm{N}} P_0 - \gamma_{\mathrm{n}} n P_+ + \gamma_{\mathrm{p}} p P_0 + \Omega(Q_0 P_0 - Q_- P_+) = -\frac{\partial P_0}{\partial t}, \quad (2)$$

where $Q_{0/-}(\boldsymbol{r},t)$ and $P_{0/-}(\boldsymbol{r},t)$ are the densities of $NV^{0/-}$ and $N^{+/0}$, respectively, $n(\boldsymbol{r},t)$ and $p(\boldsymbol{r},t)$ are the densities of free electrons and holes, respectively, $k_{0/-}[I(\boldsymbol{r}),\lambda]$ and $k_N[I(\boldsymbol{r}),\lambda]$ are the $NV^{0/-}$ and N^0 photoionization rates, respectively, which are functions of the optical intensity $I(\boldsymbol{r})$ and wavelength λ, $\kappa_{\mathrm{n/p}}$ are the electron/hole volume trapping rates of $NV^{0/-}$, $\gamma_{\mathrm{n/p}}$ are the electron/hole volume trapping rates of $N^{+/0}$, and Ω is the volume tunneling rate of electrons between NV and N centers that determines the defect charge states in thermal equilibrium.

Conservation of charge and of the approximately homogeneous concentrations of NV (Q) and N (P) centers introduces the constraints

$$\int_V \rho(\boldsymbol{r},t) dr^3 = 0, \;\; Q_0 + Q_- = Q, \;\; P_+ + P_0 = P \quad (3)$$

where $\rho = e(P_+ - Q_- + p - n)$ is the net charge density, e is the fundamental charge and the

integral is over the volume of the diamond V. Note that these constraints combined with the master equations imply that in thermal equilibrium $Q_-^{(\mathrm{eq})} = P_+^{(\mathrm{eq})}$and $Q_-^{(\mathrm{eq})}/Q = (1 + Q/P)^{-1}$. Furthermore, the second and third constraints in Eq. (3) can be used to reduce the four master equations to just the following two

$$\frac{\partial Q_-}{\partial t} = (k_0 + \kappa_\mathrm{n} n)Q - \left(k_0 + k_- + \kappa_\mathrm{n} n + \kappa_\mathrm{p} p\right)Q_- + \Omega[Q(P - P_+) - Q_- P],$$
$$\frac{\partial P_+}{\partial t} = \left(k_\mathrm{N} + \gamma_\mathrm{p} p\right)P - \left(k_\mathrm{N} + \gamma_\mathrm{n} n + \gamma_\mathrm{p} p\right)P_+ + \Omega[Q(P - P_+) - Q_- P]. \quad (4)$$

iii-Carrier diffusion and trapping

Assuming drift-diffusion transport, the free electron and hole diffusion equations are

$$\frac{\partial n}{\partial t} = \boldsymbol{\nabla} \cdot (\mu_\mathrm{n} n\boldsymbol{E} + D_\mathrm{n}\boldsymbol{\nabla} n) + k_- Q_- + k_\mathrm{N} P_0 - \kappa_\mathrm{n} n Q_0 - \gamma_\mathrm{n} n P_+ \ ,$$
$$\frac{\partial p}{\partial t} = -\boldsymbol{\nabla} \cdot \left(\mu_\mathrm{p} p\boldsymbol{E} - D_\mathrm{p}\boldsymbol{\nabla} p\right) + k_0 Q_0 - \kappa_\mathrm{p} p Q_- - \gamma_\mathrm{p} p P_0 \ , \quad (5)$$

where $\mu_\mathrm{n} \sim 2.15 \times 10^{11}$ µm^2/Vs and $\mu_\mathrm{p} \sim 1.70 \times 10^{11}$ µm^2/Vs are the approximately homogeneous and isotropic electron and hole mobilities in type-1b diamond[11], respectively, $D_\mathrm{n} = \mu_\mathrm{n} k_B T/e \sim 5.5$ µm^2/ns and $D_\mathrm{p} = \mu_\mathrm{p} k_\mathrm{B} T/e \sim 4.3$ µm^2/ns are the electron and hole diffusion constants at room temperature, respectively, k_B is Boltzmann's constant, T is the temperature. Finally

$$\boldsymbol{E}(\boldsymbol{r}) = -\boldsymbol{\nabla} \int_V \frac{\rho(\boldsymbol{r}', t)}{4\pi\varepsilon|\boldsymbol{r} - \boldsymbol{r}'|} dr'^3 \quad (6)$$

is, in the absence of an applied electric field, the electric field created by the separation of charge in the diamond and ε is the permittivity of diamond.

Applying the last two constraints in Supplementary Eq. (3), the diffusion equations become

$$\frac{\partial n}{\partial t} = \boldsymbol{\nabla} \cdot (\mu_\mathrm{n} n\boldsymbol{E} + D_\mathrm{n}\boldsymbol{\nabla} n) + k_- Q_- + k_\mathrm{N}(P - P_+) - \kappa_\mathrm{n} n(Q - Q_-) - \gamma_\mathrm{n} n P_+ \ ,$$
$$\frac{\partial p}{\partial t} = -\boldsymbol{\nabla} \cdot \left(\mu_\mathrm{p} p\boldsymbol{E} - D_\mathrm{p}\boldsymbol{\nabla} p\right) + k_0(Q - Q_-) - \kappa_\mathrm{p} p Q_- - \gamma_\mathrm{p} p(P - P_+) \ , \quad (7)$$

Thus, the combination of Supplementary Eqs. (4) and (7) define the four equations of the complete model of defect photoionization and charge diffusion. In order to establish the boundary conditions of the model, we apply the first constraint defined in Supplementary Eq. (3)

and, in so doing, we find

$$\frac{\partial}{\partial t}\int_V (P_+ - Q_- + p - n)dr^3 = -\int_V \left[\nabla\cdot\left(\mu_p p\boldsymbol{E} - D_p\nabla p\right) + \nabla\cdot\left(\mu_n n\boldsymbol{E} + D_n\nabla n\right)\right]dr^3 = 0$$

$$\rightarrow \oint_{\partial V}\left(\mu_p p\boldsymbol{E} - D_p\nabla p\right)\cdot\hat{\boldsymbol{s}}dr^2 + \oint_{\partial V}\left(\mu_n n\boldsymbol{E} + D_n\nabla n\right)\cdot\hat{\boldsymbol{s}}dr^2 = 0\ ,$$

where in the last step, Gauss' integral theorem has been applied to transform the volume integrals into surface integrals over the closed surface ∂V of the volume V with unit outwards normal vector $\hat{\boldsymbol{s}}$. If the defect photoionization and charge diffusion occurs in a region far from the surface of the diamond, then $n\sim 0$ and $p\sim 0$ on ∂V. This, in turn, implies that $\nabla \mathrm{n}\cdot\hat{\boldsymbol{s}} = 0$ and $\nabla \mathrm{p}\cdot\hat{\boldsymbol{s}} = 0$ must also vanish on ∂V, thereby establishing the complete set of boundary conditions

$$n|_{\partial V} = 0,\qquad p|_{\partial V} = 0,\qquad \nabla n\cdot\hat{\boldsymbol{s}}|_{\partial V} = 0,\qquad \nabla p\cdot\hat{\boldsymbol{s}}|_{\partial V} = 0. \tag{8}$$

The model equations are solved by combining these boundary conditions with the following initial conditions

$$Q_-(\boldsymbol{r},0) = Q_-^0(\boldsymbol{r}),\qquad P_+(\boldsymbol{r},0) = Q_-^0(\boldsymbol{r}),\qquad n(\boldsymbol{r},0) = 0,\qquad p(\boldsymbol{r},0) = 0, \tag{9}$$

which define the initial (possibly inhomogeneous) densities of NV^- and N^+ centers and assume that there are initially no free electrons or holes.

To carry out our calculations, we assume cylindrical symmetry and dominant diffusion forces [i.e., $D_n\nabla^2 n \gg \mu_n\nabla\cdot(n\boldsymbol{E})$] (see last subsection in Supplementary Note 2). The latter allows us to ignore electric forces and avoid the computational complexity they introduce by being functionals of the net charge density [as defined in Supplementary Eq. (6)]. These two assumptions allow us to recast the set of four master equations as

$$\frac{\partial Q_-}{\partial t} = (k_0 + \kappa_n n)Q - \left(k_0 + k_- + \kappa_n n + \kappa_p p\right)Q_- + \Omega[Q(P - P_+) - Q_- P]\ ,$$

$$\frac{\partial P_+}{\partial t} = \left(k_N + \gamma_p p\right)P - \left(k_N + \gamma_n n + \gamma_p p\right)P_+ + \Omega[Q(P - P_+) - Q_- P]\ ,$$

$$\frac{\partial n}{\partial t} = D_n\nabla^2 n + k_- Q_- + k_N(P - P_+) - \kappa_n n(Q - Q_-) - \gamma_n n P_+\ ,$$

$$\frac{\partial p}{\partial t} = D_p\nabla^2 p + k_0(Q - Q_-) - \kappa_p p Q_- - \gamma_p p(P - P_+)\ , \tag{10}$$

where ∇^2 is now the Laplace operator in the two-dimensions of the observed photoionization and charge diffusion patterns.

Supplementary Note 2 | Numerical simulations

In this section, we provide the details of the simulations presented in the main text. As discussed above, the complete defect photoionization and charge diffusion model contains many parameters that are unknown and must be estimated. We begin by discussing our parameter estimations. We then describe the method of our simulations and our results for red and green excitations.

Note that due to the large number of parameters, we have not aimed to quantitatively simulate our observations, but to rather qualitatively demonstrate that we have identified, and our model describes, the main physical mechanisms at play. We leave it to future systematic measurements to precisely determine the parameters that quantify each mechanism.

i-Parameter estimation

The parameters to estimate are: P, Q, Ω, k_0, k_-, k_N, κ_n, κ_p, γ_n and γ_p. As stated in the main text IR absorption spectroscopy indicates a concentration of neutral nitrogen impurities of 40 ± 4 ppm$\sim(70 \pm 7) \times 10^5$ μm^{-3}, which here we assume as representative of the total nitrogen concentration P. Based on the known correlation between the nitrogen and NV concentration in CVD diamond[13], we set the total NV concentration at $Q \sim 10^{-2} P \sim 0.4$ ppm or ~0.7×10^5 μm^{-3}.

It is difficult to estimate beforehand the NV photo-ionization rates because prior studies have focused on single NVs where the laser power used (of order 1 μW) is much lower than in the present experiments[4]. The ionization rates grow quadratically in the limit of low laser powers but one anticipates a linear growth once the illumination intensity is sufficiently strong to saturate the NV first excited state. With these considerations in mind, a lower bound for the NV photoionization rate would be ~100 kHz for green light illumination; a much lower value is expected for red-light-induced photoionization. Given that photoconductivity measurements appear to imply that the N^0 photoionization rate under red excitation is much smaller than that under green excitation and is also small compared to NV^-, we expect that $k_\mathrm{N} \ll k_-$ under red excitation. Observations of the recovery to the thermal equilibrium distribution of defect charge states in the dark indicates that $\Omega[Q(P - P_+) - Q_- P] \ll 1$ Hz, and so can be safely ignored in our calculations of the much faster photoionization and diffusion dynamics.

The electron and hole trapping rates of the N center may be estimated using the

expressions

$$\gamma_{\rm n} = \sigma_{\rm n}[{\rm N}^+]\langle v_{\rm n}\rangle \approx \sigma_{\rm n}[{\rm N}^+]\sqrt{\frac{k_{\rm B}T}{m_n}},$$

$$\gamma_{\rm p} = \sigma_{\rm p}[{\rm N}^0]\langle v_{\rm p}\rangle \approx \sigma_{\rm p}[{\rm N}^0]\sqrt{\frac{k_{\rm B}T}{m_{\rm p}}}\ , \qquad (11)$$

where $\sigma_{\rm n/p}[N^{+/0}]$ is the electron/hole trapping cross-sections of the N center, $v_{\rm n/p}$ is electron/hole thermal velocity and $m_{\rm n/p}$ is the electron/hole effective mass. Since diamond is an anisotropic valley conductor, the electron/hole effective mass is different for longitudinal ($m_{\rm n}$~$1.15\ m_{\rm e}$, $m_{\rm p}$~$1.1\ m_e$, where $m_{\rm e}$ is the free electron mass) and transverse ($m_{\rm n}$~$0.22\ m_e$, $m_{\rm p}$~$0.3\ m_{\rm e}$) wavevectors (relative to valley axis)[14]. For the sake of the calculations considered here, we have made the simplification that the effective masses of electrons and holes are isotropic and equal to $m_{\rm n}$~$m_{\rm p}$~$m_{\rm e}$. Using the measurements[10,11] $\sigma_{\rm n}[{\rm N}^+]$~$3\times10^{-6}\,\mu{\rm m}^2$ and $\sigma_p[{\rm N}^0]$~1×10^{-8} μm^2, we therefore expect that at room temperature $\gamma_{\rm n}$~300 kHz μm^3 and $\gamma_{\rm p}$~0.6 kHz μm^3.

Since the NV center is a deeper trap than the N center, it is expected that $\kappa_{\rm n} < \gamma_{\rm n}$. Furthermore, since NV$^-$ is an attractively charged trap for holes, whereas NV^0 is a neutral trap of electrons, one anticipates $\kappa_{\rm p} > \kappa_{\rm n}$.

Our numerical modeling starts with the observations of Figs. 2, 3b (upper row), and 5 to determine all ionization rates (k_-, k_0, $k_{\rm N}$) under 2 mW and 1.8 mW of red or green, fixed-point illumination, respectively. These values are subsequently used as a reference and correspondingly scaled for different illumination intensities. This scaling is made quadratic or linear to reflect the two-photon or one-photon processes governing NV and nitrogen ionization, respectively. We make use of this scaling to model the effect of the red laser scan (last step in the Sequence of Fig. 3a) on the green-induced NV$^-$ patterns (lower row in Fig. 3b) as well as the observed NV$^-$ maps on the split background (Fig. 4), see Supplementary Table 1.

ii-Red excitation

In principle, no free holes are created under red excitation because NV^0 cannot be directly photo-ionized. However, calculations based on an 'electron-only' model (i.e., $k_0(\lambda = 632\ {\rm nm}) = 0$) are inadequate to reproduce our observations. An example is presented in

Supplementary Fig. 4 where we calculate the pattern emerging from 10 s of fixed-point illumination with red light: The image corresponding to $k_0/k_- = 0$ (lower graph in the set) shows reasonable agreement with experiment on the pre-bleached, right half of the crystal, but, unlike the experiment (upper left corner), produces a bright torus above the non-bleached background on the left half. The latter could be anticipated because the exclusive production of electrons from photo-ionized NV^- centres can only increase the NV^- population in the immediate vicinity of the beam, in contrast with the observations in Figs. 1 and 4b.

An 'electron-only' model, however, must be understood as an approximation because a variety of processes are likely to enable a low-rate back conversion from NV^0 to NV^-. As explained in the main text, even a small hole production rate has a significant effect given the very low hole-trapping efficiency of nitrogen (Supplementary Table 1). Good agreement is attained with $k_0/k_- \cong 0.05$ (center image in Supplementary Fig. 4); note that higher ratios lead to unobserved behaviour (upper image in Supplementary Fig. 4).

The nature of the process at play is difficult to identify at present: Comparison of similarly-sized patterns from different combinations of laser power and exposure time (e.g., 2 mW for ~10 s versus 1 mW for ~25 s in Supplementary Fig. 6a) reveals comparable NV^- concentrations both in the bright and dark sections of the resulting ring-like structures. We surmise, therefore, that the electron and hole production rates scale similarly with light intensity suggesting that hole-production under red excitation is also the result of a two-photon process (at least within the range of laser intensities investigated in this study). Additional work, however, will be required to ascertain whether this process originates from NV^0 back-conversion to NV^- (as modeled herein) or from dynamic charge inter-conversion of some other, lower-concentration defect in our diamond crystal.

While a comprehensive description of the effect of each parameter on the simulated pattern is impractical, additional insight on the model accuracy can be attained by examining the model sensitivity to two key variables, namely, the electron and hole trapping rates. For example, a brighter-than-background torus is generated when κ_{n} is made ten-fold larger (or when κ_{p} is made ten-fold smaller) than the value listed in Supplementary Table 1 (central image). In the opposite regime, a fully bleached disk results when κ_{n} (κ_{p}) is made ten-fold smaller (larger) than the optimum, a consequence of the enhanced capture of holes by the NVs. Note that these non-optimum values also produce patterns extending over an area larger (or

smaller) than that observed.

Although our modeled patterns are sensitive to relatively small (e.g., 20%) changes of *individual* parameters, we find a considerably broader error margin if several parameters are modified simultaneously. For example, we find moderate agreement with experiment using alternative parameter sets where the ratios between all ionizing rates are kept constant. These alternate 'optimal' sets, however, are not dramatically different from each other, and typically correspond to *correlated* parameter changes by a factor 2 to 4. The error margins listed in Supplementary Table 1 reflect the range within parameter space containing these alternate 'optimal' sets. We emphasize, however, that best overall agreement is attained with the 'main' parameter set as listed.

While throughout our modeling we kept constant the nitrogen and NV concentrations, a question of interest is how local, mesoscale changes — by as much as a factor ~2 over distances of a few microns — influence the charge dynamics. In general, we find that separately changing P or Q leads to the appearance of pattern features not observed experimentally, even if a new "optimal" set of parameters is identified. The nitrogen ionization rate k_N proved to be particularly sensitive to these changes, which can be qualitatively understood from the key role played by nitrogen in defining the pattern size and growth rate under red excitation. On the other hand, comparison between calculated NV^- patterns where both P and Q are equally scaled (e.g., P and Q double while Q/P as well as all other parameters take the values listed in Supplementary Table 1) shows that, except for a moderate size change, most pattern features remain unaffected. These findings are consistent with the well-defined symmetry of the observed patterns, seen to be insensitive to local changes in the impurity content (see, e.g., Figs. 4, 5, and 6 in the main text).

iii-Green excitation

Under green excitation, free holes are created by the photoionization of NV^0 at a rate $k_0(\lambda = 532\text{ nm})$, here found to take an approximate value equal to $\sim 2k_-(\lambda = 532\text{ nm})$, in qualitative agreement with prior observations[4]. Both k_0 and k_- take absolute values — 3.5 MHz and 1.6 MHz, respectively — in reasonable agreement with the extrapolated absolute rates previously observed in single NV centers[4] (respectively ~2.7 MHz and ~1.2 MHz when we adapt the illumination conditions — 1 μW over a ~300 nm diameter spot — to those used herein). Note

that all other parameters remain unchanged relative to those used for red illumination, including the NV electron and hole photo-ionization rates κ_{n} and κ_{p}, whose exact values have, as expected, a large impact on the calculated model (left and right two-dimensional graphs in the center image of Supplementary Fig. 5). As discussed in the main text, the listed set of parameters also yields pattern growth rates in qualitative agreement with the experiment, both for red (Fig. 2g) and green (Fig. 5h) illumination (see also Supplementary Fig. 6). Overall, however, we calculate slower rates than observed experimentally, a trend that persists regardless the exact values assigned to the ionization and trapping constants. Faster growth can be attained via a slight increase of the electron and hole diffusion constants — otherwise set to fixed values, see subsection (iii) in Supplementary Note 1 and Supplementary Table 1. The latter would correspond to slightly greater electron and hole mobilities in the present sample, a possible scenario given the impact of impurities on the final mobility value[15].

Comparison of the nitrogen photo-ionization rate k_N under green and red illumination shows a reduction by six orders of magnitude, from 27 kHz at 532 nm to 27×10^{-3} Hz at 632 nm for our present illumination conditions. This sharp decrease is partially consistent with prior observations[9], though it suggests that the onset of nitrogen photo-ionization takes place at about 2.1 eV, somewhat lower than the value reported in Supplementary Ref. [9] (2.2 eV). More recent photo-ionization experiments in nitrogen-rich samples[7] set the onset at 1.9 eV making us think that the exact value may be a function of the nitrogen content (much lower in Supplementary Ref. [9] than in Supplementary Ref. [7]). Therefore, additional, quantitative experiments (i.e., using a calibrated set of samples with a varying nitrogen concentration) will be necessary to clarify this point.

Similar to subsection (ii), we find that alternate parameter sets can display moderate agreement with experiment if all photo-ionization rates are changed simultaneously so that the pair ratios remain constant. As for red illumination, the parameter subspace where modeling and observations coincide is confined, with separate 'optimal' sets requiring correlated parameter changes rarely exceeding a factor 3-4. By far, best overall agreement *for green and red illumination* is attained with the set of 'main' values listed in Supplementary Table 1. Along the same lines, we find that a moderate, equal scaling of P and Q — otherwise kept constant throughout our modeling — does not introduce major changes in the calculated NV^- patterns. By contrast, separately altering either impurity content (e.g., by a factor 2 to 4) does require a new

parameter set (and typically results in a poorer agreement with experiment). Unlike the case of red excitation (see last paragraph in Section (ii) above), we find that all corrections in the parameter set tend to be commensurate with the change introduced in P or Q.

iv-Drift versus diffusion versus trapping

To investigate the influence of electrostatic fields on the dynamics of photo-ionized carriers, we start by considering the quasi-steady state where the fluorescence pattern is only changing near the expanding outer radius. Limiting for now our analysis to red excitation, the radial charge distribution in two dimensions is crudely given by,

$$\rho(r) = \frac{\varrho_l}{\pi}\begin{cases} \dfrac{1}{R_1^2} \ , & r \leq R_1 \\ \dfrac{-1}{R_2^2 - R_1^2} \ , & R_1 < r \leq R_2 \end{cases} \tag{12}$$

where R_1 and R_2 respectively represent the inner and outer radii in the red-induced pattern (see Fig. 2), and we assume that the inner region under optical excitation has gained a uniform positive charge per unit length ϱ_l. We also assume that the fluorescent ring features an equally uniform (but negative) charge distribution so that the total net charge is null. The radial electric field produced by this charge distribution is

$$E(r) = \frac{\varrho_l}{2\pi\varepsilon}\begin{cases} \dfrac{r}{R_1^2} \ , & r \leq R_1 \\ \dfrac{1}{r}\dfrac{R_2^2 - r^2}{R_2^2 - R_1^2} \ , & R_1 < r \leq R_2 \end{cases} \tag{13}$$

where ε is the dielectric constant of diamond. To gauge the effect of the electrostatic field on the carrier dynamics we consider the ratio ϕ between the diffusion and drift forces acting on the free electron density n. From Supplementary Eq. (7) we find

$$\phi = \frac{D_n}{\mu_n E}\frac{\partial_r n}{n} \quad . \tag{14}$$

In the limit of a pure diffusion solution (where $n \propto exp(-r^2/4D_n t)$), Supplementary Eq. (14) yields

$$\phi = -\frac{\pi\varepsilon R_1^2}{\mu_n \varrho_l t}\begin{cases} 1 \ , & r \leq R_1 \\ \dfrac{r^2}{R_1^2}\dfrac{(R_2^2 - R_1^2)}{(R_2^2 - r^2)} \ , & R_1 < r \leq R_2 \end{cases} \quad . \tag{15}$$

At $r \sim R_1$ we thus find the minimum value $|\phi|_{min} \sim \frac{\pi \varepsilon R_1^2}{\mu_n \varrho_l t}$. Setting t to 5 ns — the time needed for the electron to diffuse away from the inner ring — and defining e as the electronic charge, we conclude that for $|\phi|_{min}$ to drop below 1 the normalized charge per unit length ϱ_l / e must be greater than 10^6 µm^{-3}. Given the nitrogen concentration $P = 40$ ppm and considering an optical spot with transverse dimensions of 1×1 µm^2 we conclude carrier transport becomes drift-dominated when the nitrogen ionization approximately equals or exceeds 10%. As seen in Fig. 6d in the main text, the concentration of positively charged nitrogen does not exceed 0.4 ppm, suggesting that electrostatic fields do not have a major impact on the carrier transport dynamics under the present conditions.

To gain a more quantitative understanding we explicitly calculate the net charge density and electrostatic field resulting from 10 s of red (and green) illumination (Supplementary Fig. 7). Here we follow a self-consistent approach were the drift force is initially neglected from the master equations. As expected, we find that the electrostatic field reaches its maximum within the inner section of the fluorescence ring (Supplementary Fig. 7c). The electric field is substantially stronger for green illumination, a consequence of the relatively more efficient ionization of nitrogen at this wavelength. The results also show that the magnitude of the diffusion/drift ratio $|\phi(r)|$ consistently takes values larger than unity throughout the pattern, thus confirming the dominance of diffusion under the present experimental conditions. It is worth mentioning that unlike the estimate for $|\phi|_{min}$ above, this time no assumption is made on the spatial distribution of free electrons, which results from the (drift-free) master equations.

Finally, we gain further insight in the hierarchy of terms in Supplementary Eq. (7) by separately calculating diffusion and capture terms. The results are presented in Supplementary Fig. (7d) for 10 s of red (left) or green (right) illumination. In both cases, we find that trapping terms are dominant, consistent with the notion of a ‘reaction-diffusion’ regime where the pattern boundary progresses once an equilibrium concentration of charged defects is reached (see main text).

Supplementary References

[1] Liang, Z.Z. et al Synthesis of HPHT diamond containing high concentrations of nitrogen impurities using NaN_3 as dopant in metalcarbon system, *Diam. Rel. Mater.* **14**, 1932-1935 (2005).

[2] Doherty, M.W. et al. The nitrogen-vacancy colour centre in diamond, *Phys. Rep.* **528**, 1-46 (2013).

[3] Waldherr, G. Dark States of Single Nitrogen-Vacancy Centers in Diamond Unraveled by Single Shot NMR, *Phys. Rev. Lett*. **106**, 157601 (2011).

[4] Aslam, N., Waldherr, G., Neumann, P., Jelezko, F., Wrachtrup, J. Photo-induced ionization dynamics of the nitrogen vacancy defect in diamond investigated by single-shot charge state detection, *New J. Phys.* **15**, 013064 (2013).

[5] A.M. Stoneham, *Theory of Defects in Solids* (Oxford University Press, Oxford, 1975).

[6] Farrer, R.G. On the substitutional nitrogen donor in diamond, *Sol. State Commun* **7**, 685-688 (1969).

[7] Heremans, F.J., Fuchs, G.D., Wang, C.F., Hanson, R., Awschalom, D.D. Generation and transport of photoexcited electrons in single-crystal diamond, *Appl. Phys. Lett.* **94**, 152102 (2009).

[8] Nesládek, M. et al. Dominant defect levels in diamond thin films: A photocurrent and electron paramagnetic resonance study, *Appl. Phys. Lett.* **72**, 3306-3308 (1998).

[9] Isberg, J., Tajani, A., Twitchen, D.J. Photoionization measurement of deep defects in single-crystalline CVD diamond using the transient-current technique, *Phys. Rev. B* **73**, 245207 (2006).

[10] Pan, L.S., Kania, D.R., Pianetta, P., Landen, O.L. Carrier density dependent photoconductivity in diamond, *Appl. Phys. Lett*. **57**, 623-625 (1990).

[11] Han S. in *Diamond: Electronic Properties and Applications*, ed. Pan, L.S. & Kania, D.R. (Springer Science, New York, 1995).

[12] Lowther, J.E. Excited states of the vacancy in diamond, *Phys. Rev. B* **48**, 11592 (1993).

[13] Edmonds, A.M. et al. Production of oriented nitrogen-vacancy color centers in synthetic diamond, *Phys. Rev. B* **86**, 035201 (2012).

[14] Isberg, J. et al. Generation, transport and detection of valley-polarized electrons in diamond, *Nat. Mater*. **12**, 760-764 (2013).

[15] Pernot, J. Hall electron mobility in diamond, *Appl. Phys. Lett.* **89**, 122111 (2006).
